# Supplementary material for: Ketogenesis is Dispensable for the Metabolic Adaptations to Caloric Restriction
Source: Aging Cell. 2025 Dec 10;25(1):e70318. doi: 10.1111/acel.70318 (PMC12741194; doi:10.1111/acel.70318)
Supplement: Supplementary file 1 — Figure S1: The ablation of ketogenesis does not affect the fat, lean, and adiposity of mice undergoing caloric restriction. (A) A schematic of our workflow. Abbreviations: GTT (glucose tolerance test), ITT (insulin tolerance test), PTT (pyruvate tolerance test), LTT (lipid tolerance test), and KTT (ketone tolerance test). (B‐E) In male mice, their food intake (B), fat mass (C), lean mass (D), and adiposity % (E) over 7 months of the caloric restriction protocol. For all male mice data presented here, at the beginning of the experiment, WT‐AL, KO‐AL, WT‐CR, and KO‐CR, n = 7, 6, 8, and 7, respectively. (F–I) In female mice, their food intake (F), fat mass (G), lean mass (H), and adiposity % (I) over 7 months of the caloric restriction protocol. For all female mice data presented here, at the beginning of the experiment, WT‐AL, KO‐AL, WT‐CR, KO‐CR, n = 6, 7, 7, 9, respectively. Data presented as mean ± SEM. Figure S2: Male metabolic health characterization. (A, B) After a 7 h fast in male mice, glucose tolerance test (A; 1 g/kg; I. P.) and insulin tolerance test (B; 0.5 U/kg; I. P.). For WT‐AL, KO‐AL, WT‐CR, and KO‐CR, glucose tolerance test n = 7,6,8,7; insulin tolerance test n = 5,5,8,6. (C) In male mice, raw blood glucose value of the pyruvate tolerance test (2 g/kg; I. P.) measured after 21 h of fasting. For WT‐AL, KO‐AL, WT‐CR, and KO‐CR, n = 7,6,8,6. (D) After 21 h of fasting in male mice, glucose‐stimulated insulin secretion (2 g/kg; I. P.), insulin, and blood glucose levels. For WT‐AL, KO‐AL, WT‐CR, and KO‐CR, insulin n = 7,7,8,8; glucose n = 9 each group. (A–D) ***p < 0.001, ****p < 0.0001, Sidak's test post 2‐way ANOVA. Data presented as mean ± SEM. Figure S3: Female metabolic health characterization. (A, B) After a 7 h fast in female mice, glucose tolerance test (A; 1 g/kg; I. P.) and insulin tolerance test (B; 0.5 U/kg; I. P.). For WT‐AL, KO‐AL, WT‐CR, and KO‐CR, glucose tolerance test n = 6,7,7,8; insulin tolerance test n = 6,6,7,8. (C) In female mice, raw [file ACEL-25-e70318-s002.pdf]

Supplemental Figure 1.

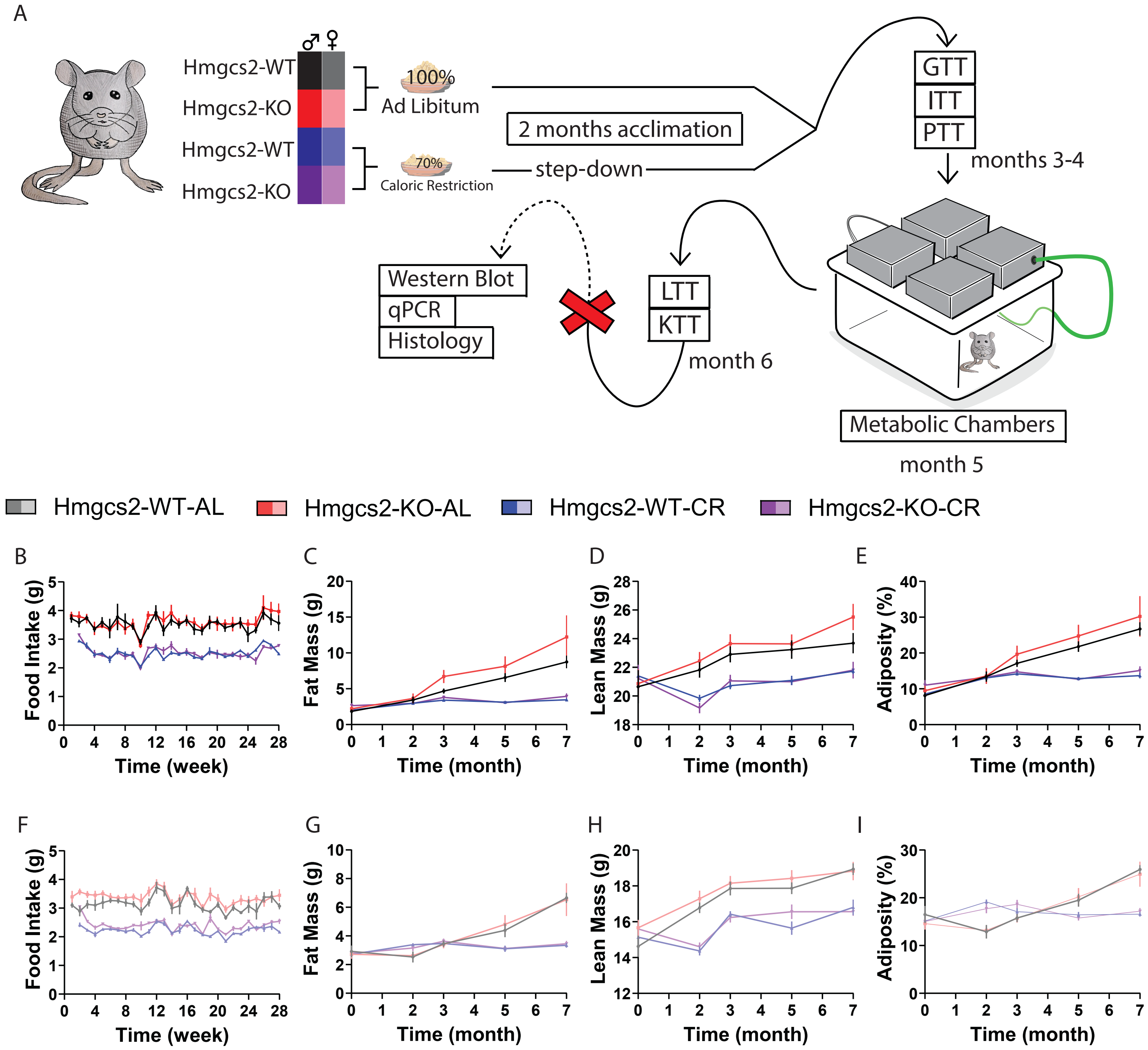

Supplemental Figure Legends

Supplemental Figure 1. The ablation of ketogenesis does not affect the fat, lean, and adiposity of mice undergoing caloric restriction.

(A) A schematic of our workflow. Abbreviations: GTT (glucose tolerance test), ITT (insulin tolerance test), PTT (pyruvate tolerance test), LTT (lipid tolerance test), and KTT (ketone tolerance test). **(B-E)** In male mice, their food intake (B), fat mass (C), lean mass (D), and adiposity % (E) over 7 months of the caloric restriction protocol. For all male mice data presented here, at the beginning of the experiment, WT-AL, KO-AL, WT-CR, KO-CR, n=7, 6, 8, and 7 respectively. **(F-I)** In female mice, their food intake (F), fat mass (G), lean mass (H), and adiposity % (I) over 7 months of the caloric restriction protocol. For all female mice data presented here, at the beginning of the experiment, WT-AL, KO-AL, WT-CR, KO-CR, n=6, 7, 7, 9 respectively. Data presented as mean ± SEM.

Supplemental Figure 2.

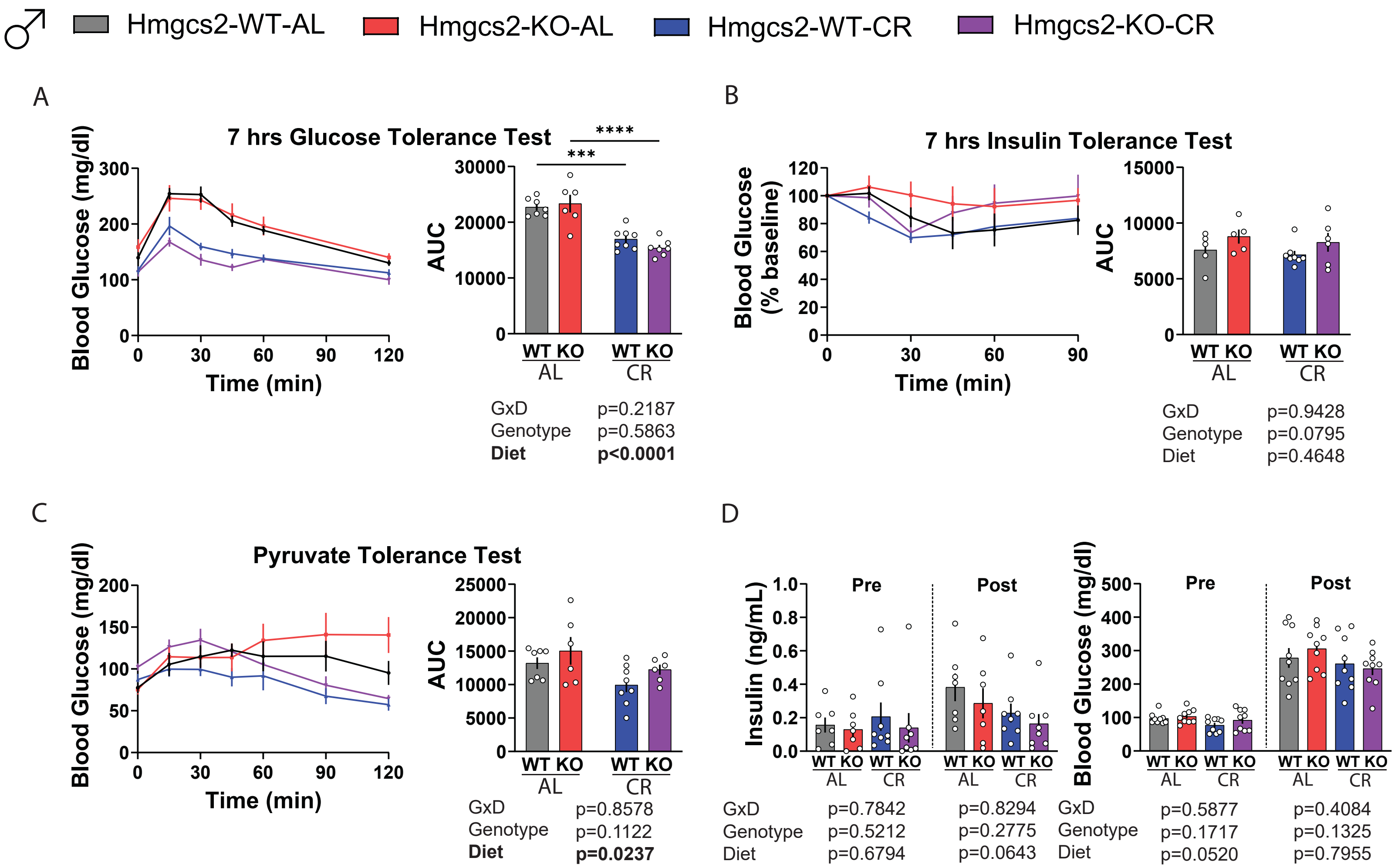

Supplemental Figure 2. Male metabolic health characterization

(A-B) After 7 hrs fast in male mice, glucose tolerance test (A; 1 g/kg; I. P.) and insulin tolerance test (B; 0.5 U/kg; I. P.). For WT-AL, KO-AL, WT-CR, and KO-CR, glucose tolerance test n=7,6,8,7; insulin tolerance test n=5,5,8,6. **(C)** In male mice, raw blood glucose value of the pyruvate tolerance test (2 g/kg; I. P.) after 21 hrs fasting. For WT-AL, KO-AL, WT-CR, and KO-CR, n=7,6,8,6. **(D)** After 21 hrs fasting in male mice, glucose stimulated insulin secretion (2 g/kg; I. P.) insulin and blood glucose levels. For WT-AL, KO-AL, WT-CR, and KO-CR, insulin n=7,7,8,8; glucose n=9 each group. (A-D) ***$p<0.001$, ****$p<0.0001$, Sidak's test post 2-way ANOVA. Data presented as mean ± SEM.

Supplemental Figure 3.

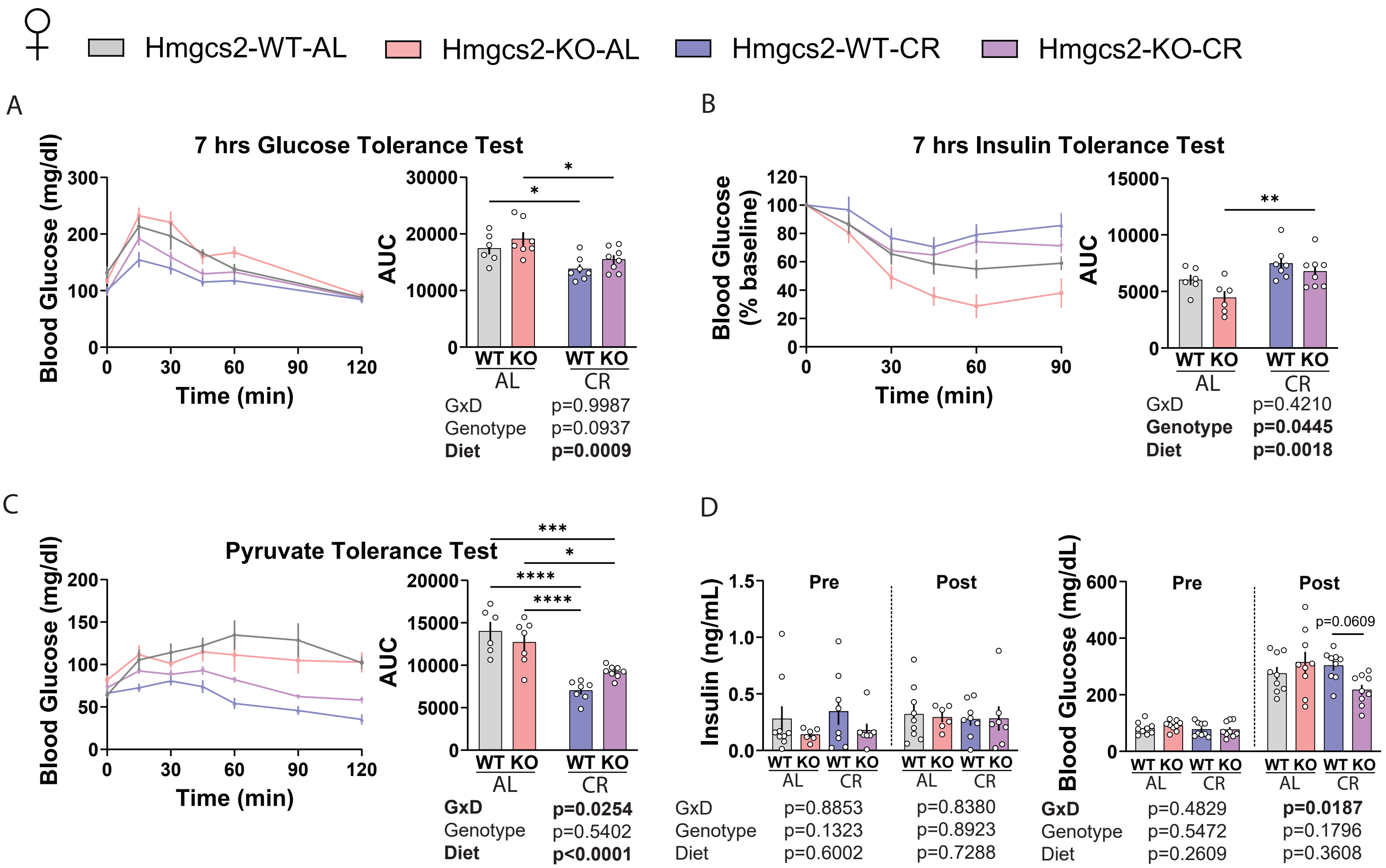

Supplemental Figure 3. Female metabolic health characterization.

(A-B) After 7 hrs fast in female mice, glucose tolerance test (A; 1 g/kg; I. P.) and insulin tolerance test (B; 0.5 U/kg; I. P.). For WT-AL, KO-AL, WT-CR, and KO-CR, glucose tolerance test n=6,7,7,8; insulin tolerance test n=6,6,7,8. **(C)** In female mice, raw blood glucose value of the pyruvate tolerance test (2 g/kg; I. P.) after 21 hrs fasting. For WT-AL, KO-AL, WT-CR, and KO-CR, n=6,7,7,8. **(D)** After 21 hrs fasting in female mice, glucose stimulated insulin secretion (2 g/kg; I. P.) insulin and blood glucose levels. For WT-AL, KO-AL, WT-CR, and KO-CR, insulin n=9,6,8,7; glucose n=9 each group. (A-D) *$p<0.05$, **$p<0.01$, ***$p<0.001$, ****$p<0.0001$, Sidak’s test post 2-way ANOVA. Data presented as mean ± SEM.

Supplemental Figure 4.

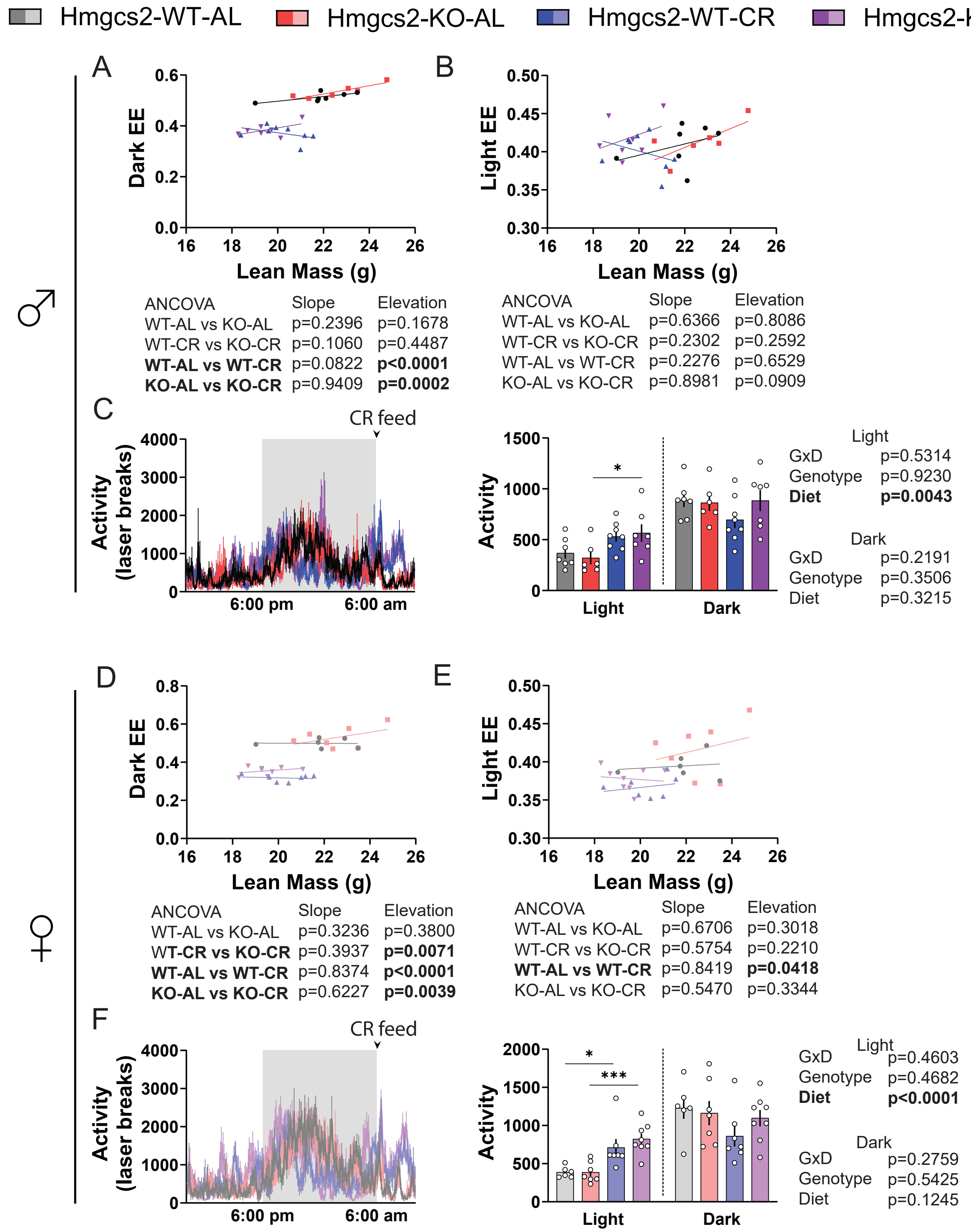

Supplemental Figure 4. ANCOVA analysis and metabolic chamber activity.

(A-C) In male mice, ANCOVA analysis of average dark phase (A) and light phase (B) energy expenditure against lean mass. (**C**) Activity during metabolic chambers test in male mice over 24 hrs and binned by averaging all data points in the light or dark cycle. For WT-AL, KO-AL, WT-CR, and KO-CR, male n=7,6,8,7. **(D-F)** In female mice, ANCOVA analysis of average dark phase (D) and light phase (E) energy expenditure against lean mass. (**F**) Activity during metabolic chambers test in female mice over 24 hrs and binned by averaging all data points in the light or dark cycle. For WT-AL, KO-AL, WT-CR, and KO-CR, female n=6,7,7,8. (C, F) *$p<0.05$, ***$p<0.001$; Sidak's test post 2-way ANOVA performed separately for each cycle. Data presented as mean ± SEM.

Supplemental Figure 5.

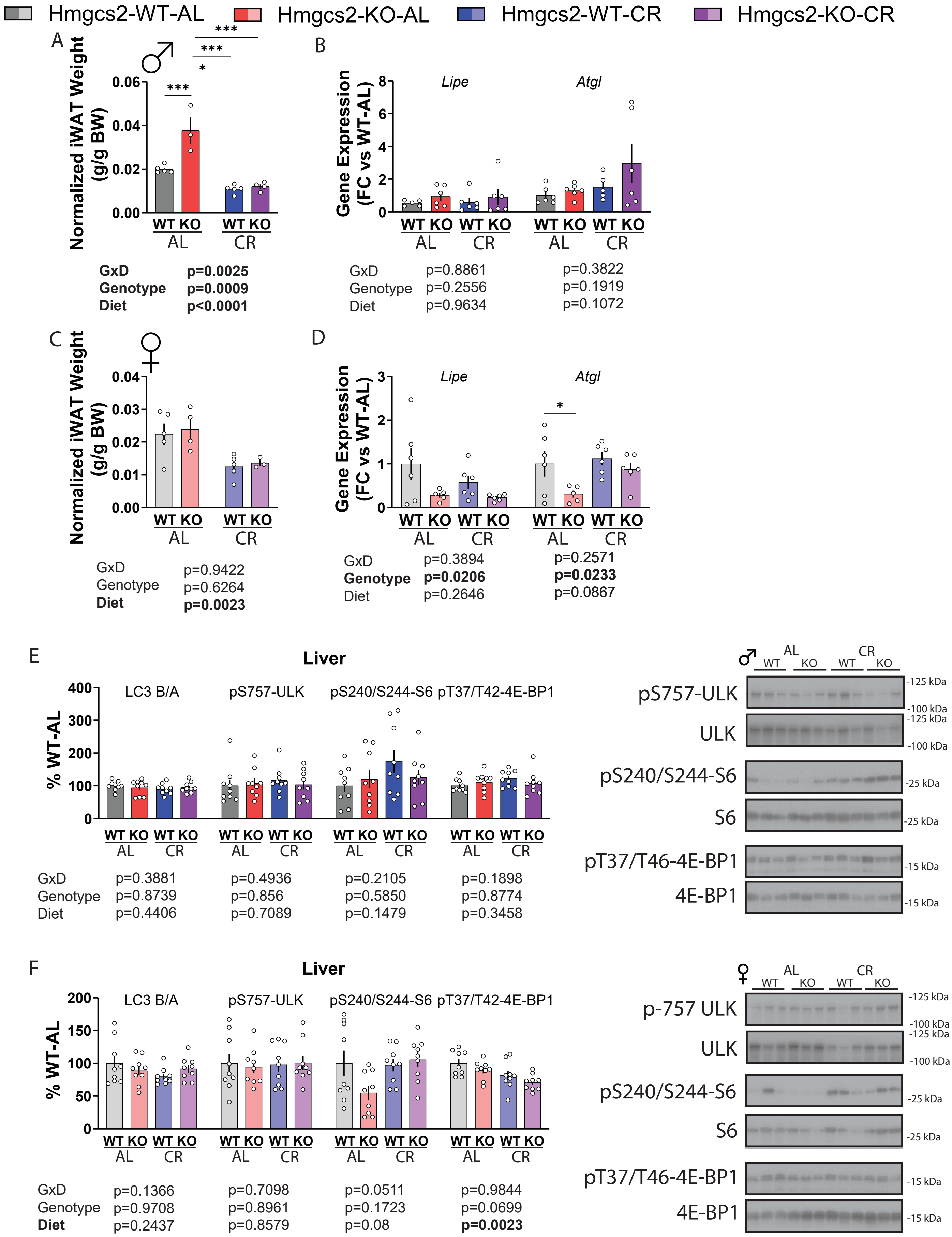

Supplemental Figure 5. Lipolytic gene expression in the iWAT and mTORC1 signaling in the liver.

(A-B) In male mice, body weight-normalized iWAT weight (A) and the expression of *Lipe* and *Atgl*. (B) in the iWAT of male mice. For WT-AL, KO-AL, WT-CR, and KO-CR, iWAT weight n=5,3,5,4; *Lipe* n=5,6,6,6; *Atgl* n=6,6,5,6. **(C-D)** In female mice, body weight-normalized iWAT weight (C) and the expression of *Lipe* and *Atgl* (D) in iWAT of female mice. For WT-AL, KO-AL, WT-CR, and KO-CR, iWAT weight n=5,4,5,3; both genes n=6,5,6,6. **(E-F)** LC3B/A ratio and mTORC1-related protein phosphorylation in male (E) and female (F) mice. n=9 each group. (A-F) *$p<0.05$; Sidak's test post 2-way ANOVA conducted separately for each gene (A-D) or protein/phosphor-residue (E-F). Data presented as mean ± SEM.

Supplemental Figure 6.

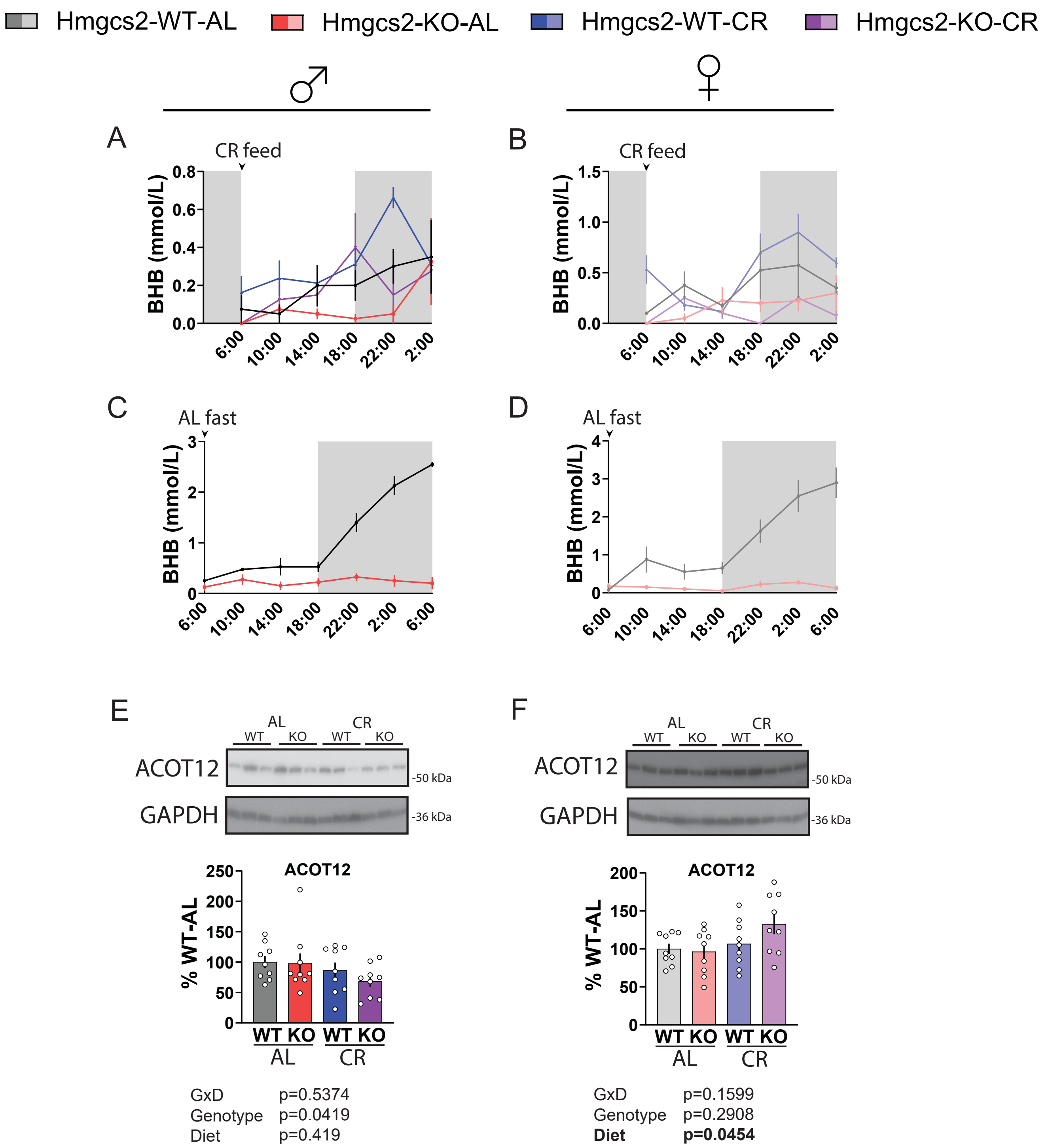

Supplemental Figure 6. Circulating βHB profile of animals during experiments and fasting; liver ACOT12 levels.

(A-B) Circulating βHB level of male (A) and female (B) mice throughout a typical day. For WT-AL, KO-AL, WT-CR, and KO-CR, male n=4,4,8,4; female n=4,4,6,4. **(C-D)** Circulating βHB level of AL-fed male (C) and female (D) mice while fasting. n=4 each group. **(E-F)** Liver western blots in male (E) and female (F) mice for ACOT12. n=9 each group. Sidak's test post 2-way ANOVA. Data presented as mean ± SEM.
